# Dangers of Raw Milk

**Published:** 1889-06-08

**Authors:** 


### WITHIN THE WARDS.

### DANGERS OF RAW MILK.

CAREFUL observers, who are by no means inclined to the creation of public "scares," are decidedly of opinion that there is a considerable degree of danger in the use of uncooked milk as food. It is believed that not only are certain exanthematous fevers communicated to consumers of raw milk, but that tubercle itself, in some of its forms, may also arise in the human subject in this way. An important paper was recently read at the Pathological Society of London, by Mr. Shattock, on "Tubercular Abscess of the Breast." In the course of the paper, it was stated that in the cow "tubercle of the udder" was a well-known disease, so much so that on the Continent its hygienic importance was generally and practically recognised. Ten or twelve years ago the minute structure of the tubercle bacillus which is found in the cow's udder was figured and described by Kolessnikow in *Virchow's Archiv.* It was found also by experiments on animals that the milk from tuberculous udders contained bacilli and was rapidly infectious. Most people are familiar with what is popularly known as "consumption of the bowels" in children. Dr. Hamilton, a distinguished Aberdeen professor, has expressed the opinion that tuberculous milk from cows may often be the cause of that distressing and fatal malady. In this connection a case is recorded of a perfectly healthy child, born of equally healthy parents, which was given to a wet-nurse to be suckled. The woman was tubercular, and the child very quickly contracted tubercular meningitis and died. The nurse's milk, on examination, was found to contain the bacilli of tubercle. The disease, tuberculosis, it is believed, can be present in an animal or a human subject without being definitely localised as an anatomical entity in any particular organ. It may therefore easily happen that a cow shall continue to be milked for months, and her milk sold as food for infants and others, before it is discovered that she is the subject of fatal and infectious disease. Certain breeds of cows are supposed to be especially liable to tubercle of the udders, and those breeds are noted for their large udders, and for the abundance of the milk which they yield. Such breeds and animals are, not unnaturally, much sought after by dairymen, and the extent of the danger is thus increased. All this sounds sufficiently alarming, but whatever conclusions may be drawn from it, one point of practical importance should certainly not be overlooked. That point has often been urged by medical men, and it must continue to be urged again and again. It is that milk should not be taken raw, but boiled. Milk needs to be cooked as much as beef or pork. Many persons, schoolboys especially, profess a strong objection to cooked milk. That is probably because no skill is exercised in the cooking. It may be cooked in half-a-dozen different ways; but two, at any rate, of these are so simple that it is inexcusable not to try them. A little sugar added to milk when boiling gives it quite a new flavour, and makes it to many boys more palatable than uncooked milk. For those who do not like what is sweet, a pinch of salt may be put in; and that, again, produces a substance having a totally different taste from plain boiled milk. Other methods of making cooked milk palatable will suggest themselves to the conscientiously careful mother or to the experienced cook. There can be no good reason why anybody should be asked to take raw milk; still less ought there to be any excuse for preferring it raw on the ground that when cooked it is less palatable.

